# Fluorinated Tryptophan Derivatives for Photo-CIDNP NMR

**DOI:** 10.1021/acs.jpcb.6c01954

**Published:** 2026-06-10

**Authors:** Anton Schmidt, Magdalena J. Faber, Audrey Ayekoi, Boris Illarionov, Adelbert Bacher, Markus Fischer, Stefan Weber

**Affiliations:** † Institute of Physical Chemistry, 9174 University of Freiburg, Freiburg 79104, Germany; ‡ Hamburg School of Food Science, Institute of Food Chemistry, 14915 University of Hamburg, Hamburg 20146, Germany; § 9184 TUM School of Natural Sciences, Garching 85748, Germany

## Abstract

Given the large hyperfine couplings commonly observed in ^19^F nuclei within radicals, fluorinated amino acids are promising candidates for investigating the solvent exposure of amino acids and redox-active cofactors in proteins, as well as for identifying photogenerated intramolecular spin-correlated radical pairs in proteins through photochemically induced dynamic nuclear polarization (photo-CIDNP) nuclear magnetic resonance (NMR). By analyzing their photo-CIDNP properties in solution, this work aims to establish the foundation for using fluorinated tryptophan derivatives in photo-CIDNP, including protein studies. Although fluorinated tyrosine derivatives have been used in several photo-CIDNP studies, there are hardly any data for fluorinated tryptophan derivatives. We assume this is due to the significant line broadening of ^19^F resonances of fluorinated tryptophan derivatives when flavin mononucleotide (FMN) is used as a photosensitizer. We attribute this to a photochemical reaction occurring between FMN and the fluorinated tryptophans. The broadening can be avoided by using fluorescein as a photosensitizer. The hyperfine couplings of ^1^H and ^19^F nuclei in the fluorinated tryptophan radicals of the commercially available derivatives 4-fluorotryptophan, 5-fluorotryptophan, 6-fluorotryptophan, and 7-fluorotryptophan were probed by geminate photo-CIDNP. Time-resolved photo-CIDNP was used to investigate the microsecond kinetics of ^19^F and ^1^H photo-CIDNP polarization in the 6-fluorotryptophan radical, revealing strongly different time evolutions due to the nuclei’s distinct paramagnetic relaxation. Experimental considerations for incorporation into proteins and for photo-CIDNP of such proteins are discussed.

## Graphical abstract

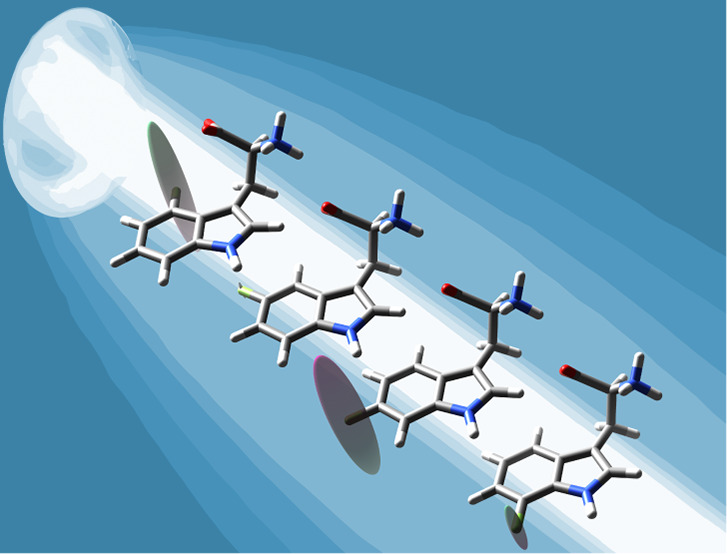

## Introduction

Photochemically induced dynamic nuclear polarization (photo-CIDNP) is a nuclear magnetic resonance (NMR) hyperpolarization technique applicable to systems that form spin-correlated radical pairs (SCRPs) as part of their photocycle.^1^ A suitable photosensitizer, usually a flavin,^2–7^ 2,2′-dipyridyl,^7–12^ fluorescein,^13–15^ or (carboxy)­benzophenone,^12,16^ is irradiated by light, and in the presence of a suitable electron donor, SCRPs are formed by electron transfer from the electron donor to the photosensitizer. Photo-CIDNP is most commonly used to enhance resonances from the aromatic amino acids tryptophan, tyrosine and histidine,^17^ which may act as electron donors. It can be applied to quantify the solvent-accessibility of amino acids in proteins,^5,7,8^ to determine intramolecular correlation times and order parameters of solvent-exposed amino acids in proteins,^9^ to study solvent-exposed flavin cofactors in flavoproteins,^18^ and to investigate folding processes on the millisecond time scale.^19^ The method is simple in terms of experimental effort, requiring only the addition of a photosensitizer and subsequent irradiation, typically by a laser or an LED. The resulting spectra are straightforward to interpret as only the resonances belonging to nuclei of the SCRP-forming moieties are hyperpolarized, whereas the thermal polarization can be canceled out prior to sample irradiation.^20^

In diffusion-controlled photo-CIDNP experiments, i.e., liquid solutions of independently diffusing electron donor and dye molecules, the isotropic hyperfine coupling constants of nuclei in the intermediate radicals largely determine the signal intensities.^12,16^ However, SCRPs are also generated in flavoproteins such as cryptochromes,^21^ photolyases,^22,23^ blue-light using FAD (BLUF) domains^24^ and mutants of light-oxygen-voltage-sensing (LOV) domains,^25,26^ as well as protein complexes, such as quinone-depleted or quinone-blocked photosynthetic reaction centers.^27–29^ In these systems, the formed radicals are unable to diffuse apart. Therefore, the hyperfine anisotropy is a crucial quantity for the magnitude of the photo-CIDNP effect in solids,^30–33^ as it is not averaged out due to the fixed orientation and distance of the radicals relative to each other. In photo-CIDNP experiments involving freely diffusing SCRPs in solution, a strong anisotropy of the hyperfine coupling can also be beneficial. Besides rotational modulation of the anisotropic Zeeman interaction, rotational modulation of the hyperfine interaction is the main source for nuclear relaxation of the involved radicals,^34^ with a quadratic dependency of the relaxation rate on the hyperfine anisotropy.^35^ For a more detailed review of the spin-sorting nature of the radical pair mechanism and the microsecond kinetics of photo-CIDNP polarization, as well as its applications to amino acids, we recommend a review by Morozova and Ivanov.^36^

Recent developments highlight the potential of hyperpolarization methods, including photo-CIDNP, in the field of protein science. For instance, 20 nM of tryptophan can be detected in just a few seconds,^14^ which is remarkable for an NMR-based method given its inherent insensitivity. In addition to its use as a hyperpolarization method, photo-CIDNP can also detect elusive SCRPs.^25,37^ Since photo-CIDNP probes nuclear hyperpolarization of ground-state molecules rather than the paramagnetic reaction intermediates themselves, the detection time shifts from the SCRP lifetime (typically in the short nanosecond range) to the nuclear transverse relaxation time of diamagnetic reaction products (typically milliseconds to seconds). Thus, photo-CIDNP can detect SCRPs with lifetimes too short for direct detection using electron paramagnetic resonance (EPR)-based methods, such as time-resolved EPR.^38^ A recent example is the 5-deazaflavin, which was historically considered a two-electron acceptor, but has been definitely proven to be capable of accepting one electron and forming a radical by using photo-CIDNP.^37^

Fluorine is a spin-1/2 nucleus with a gyromagnetic ratio similar to that of ^1^H. Combined with its natural abundance of 100% and sparse occurrence in biological samples, it is a well-established nucleus in biomolecular NMR.^39^ Its large chemical shift anisotropy (CSA) makes it sensitive to changes in its surroundings.^40^ For photo-CIDNP, a large hyperfine coupling is beneficial, since the nuclear spin sorting, and therefore hyperpolarization magnitude for a given nucleus, is based on this quantity.^41^ The high electron density of the fluorine’s 2s wave function results in a high probability of finding the unpaired electron at the position of the nucleus, leading to a high isotropic hyperfine coupling per spin population.^42^ Compared to the hydrogen 1s orbital, the same spin population leads to an approximately 37.2 times higher isotropic hyperfine coupling constant. Additionally, fluorine nuclei exhibit a large hyperfine anisotropy due to the strong localization and reduced radial extent of fluorine 2p orbitals. Overall, the strong hyperfine coupling of ^19^F nuclei in fluorine-containing radicals is expected to produce strong ^19^F photo-CIDNP.

The photo-CIDNP properties of fluorinated tyrosine derivatives have already been studied,^6,34,43^ yielding promising results for exploring the solvent-accessibility of tyrosine residues in proteins,^44^ and even for the development of MRI methods.^45,46^ 3-Fluorotyrosine is the superior choice over 2-fluorotyrosine due to its stronger hyperfine coupling of its fluorine nucleus.^35^ Conversely, fluorinated tryptophan derivatives have hardly been studied regarding their photo-CIDNP properties.^35^ Their low cost (i.e., compared to expensive ^13^C enrichment) makes them a viable option, especially when intramolecular SCRPs are involved. Fluorinated tryptophan derivatives are routinely used in spectroscopy and microscopy, including ^19^F NMR.^47^ Applications of the latter include, but are not limited to, in-cell NMR,^48,49^ determination of solvent-exposure via solvent isotope and paramagnetic effects,^50^ and tracking changes in protein conformation.^51^

We recently demonstrated that geminate ^19^F photo-CIDNP, a special case of time-resolved (tr) photo-CIDNP with no delay between laser pulse and radiofrequency pulse, can be used to determine isotropic hyperfine coupling constants of ^19^F nuclei, given that at least two fluorine nuclei are present in the SCRP.^52^ In this contribution, we aim to identify the most promising monofluorinated tryptophan derivative for ^19^F photo-CIDNP measurements in proteins. First, hyperfine mapping of ^1^H and ^19^F nuclei in 4-, 5-, 6-, and 7-fluorotryptophan, along with 8-fluoro-7,8-didemethyl flavin mononucleotide (FMN) as the photosensitizer (Figure 1), will be performed to gain insight into the electronic structure of the radicals. This will allow to determine the protonation state of the radicals in the geminate SCRP. The derivative exhibiting the strongest ^19^F photo-CIDNP enhancement under continuous-wave (cw) irradiation will then be identified, as will the more suitable photosensitizer between FMN and fluorescein. Finally, the microsecond kinetics of the photo-CIDNP polarization will be examined to investigate the longitudinal relaxation times of ^1^H and ^19^F nuclei in the 6-fluorotryptophan radicals, and the benefits of nuclei with high hyperfine anisotropies will be discussed.

**Figure 1:**
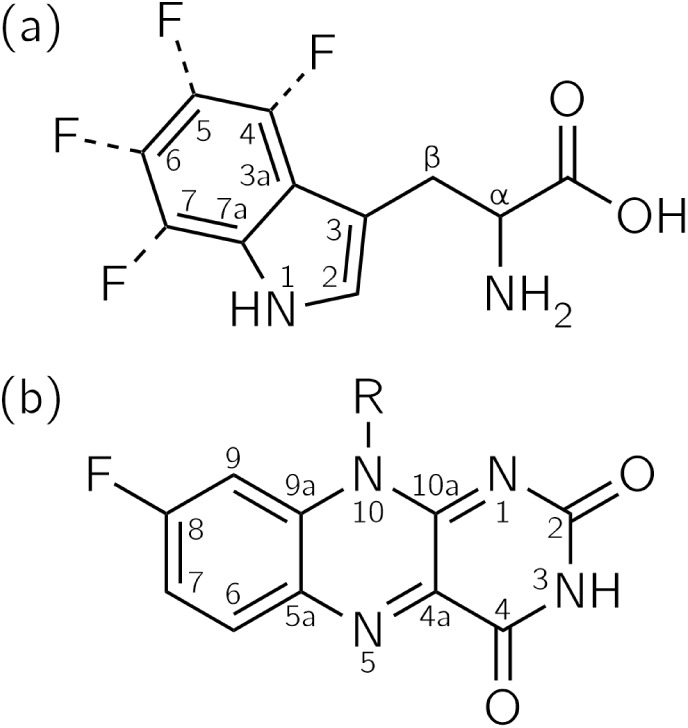
Structures of the investigated fluorinated compounds. (a) Tryptophan derivatives with fluorination at positions 4, 5, 6, or 7. (b) The isoalloxazine moiety of 8-fluoro-7,8-didemethyl-FMN. R denotes a phosphorylated ribityl side chain.

## Materials and Methods

### Sample Preparation

The synthesis and characterization of 8-fluoro-7,8-didemethyl-FMN have been described elsewhere.^52^ 8-Fluoro-7,8-didemethyl-FMN was purified via high-performance liquid chromatography (HPLC) prior to measurement. Racemic dl mixtures of 4-fluorotryptophan, 6-fluorotryptophan and 2-fluorotyrosine, as well as l-isomers of 7-fluorotryptophan and 3-fluorotyrosine, were purchased from Aaron Chemicals (San Diego, CA, USA), and 5-fluoro-dl-tryptophan was purchased from Acros Organics (Geel, Belgium). FMN and fluorescein were acquired from Sigma-Aldrich (St. Louis, MO, USA) and used without further purification.

Photo-CIDNP samples contained 1 or 2 mM of the respective fluorinated amino acid in D_2_O (99.95 atom-% D, Deutero GmbH, Kastellaun, Germany). Samples used in tr-photo-CIDNP contained either 8-fluoro-7,8-didemethyl-FMN or FMN with an absorbance of 2.5 at the long-wavelength absorbance maximum (425 and 445 nm, respectively), corresponding to concentrations of 200 μM. Samples used in cw-photo-CIDNP contained either FMN or fluorescein with an absorbance of 2.5 at the emission wavelength of the cw-laser (445 nm), corresponding to concentrations of 200 μM (FMN) and 100 μM (fluorescein).

### NMR Spectroscopy

Unless otherwise specified, ^1^H NMR experiments were performed on an Avance III HD NMR spectrometer (Bruker BioSpin GmbH, Ettlingen, Germany) operating at 14.10 T, resulting in resonance frequencies of 600 MHz for ^1^H. ^1^H NMR measurements were performed with a triple-resonance (TXI) probe. ^19^F NMR experiments were performed on an Avance Neo NMR spectrometer (Bruker BioSpin GmbH, Ettlingen, Germany) operating at 7.05 T, resulting in resonance frequencies of 282 MHz for ^19^F. ^19^F measurements were performed with a broad-band (BBFO) probe. The receiver offset was set in the middle of the two resonances to ensure uniform excitation when two resonances were recorded (whenever 8-fluoro-7,8-didemethyl-FMN was used). Otherwise, it was set on-resonance. For ^1^H NMR, the receiver offset was set to the resonance frequency of water.

In tr-photo-CIDNP, light excitation was carried out using a nanosecond-pulsed laser system, consisting of an optical parametric oscillator (OPO) (OPO Plus, Continuum, Milpitas, CA, USA) which was pumped by an Nd:YAG laser (Surelite II, Continuum, Milpitas, CA, USA), resulting in a laser pulse length of 4–7 ns. The OPO output wavelength was set to 430 nm for measurements involving 8-fluoro-7,8-didemethyl-FMN, and to 445 nm for measurements involving FMN or fluorescein. The output was coupled into an optical fiber with a diameter of 1 mm (Thorlabs, Newton, NJ, USA), and inserted into the sample via a coaxial insert (Wilmad WGS-5L), resulting in output powers of 20–21 mJ at the fiber tip. In cw-photo-CIDNP, a DHOM-H-445 cw-laser (Ultralasers, Newmarket, Canada), operating at 445 nm, was used. The laser output was coupled into an optical fiber with the same characteristics as in tr-photo-CIDNP, resulting in output powers of 300–400 mW at the fiber tip.

Tr-photo-CIDNP spectra were recorded using a presaturation pulse train designed to cancel out thermal polarization prior to optical excitation.^20^ Additionally, a WALTZ-16 sequence was implemented to decouple ^1^H in ^19^F photo-CIDNP.^53^ For acquisition of ^1^H tr-photo-CIDNP and ^19^F cw-photo-CIDNP, a destructive phase cycle with irradiation in every other scan was applied. The following sampling pulse lengths were used: 2.5 μs (for geminate tr-photo-CIDNP), 1 μs (for measuring microsecond kinetics via tr-photo-CIDNP) and 7.7–7.8 μs (for cw-photo-CIDNP; corresponding to the 90° pulse length). For measuring microsecond kinetics, a fresh sample was used for each measurement. For geminate ^1^H and ^19^F tr-photo-CIDNP experiments, 128 scans and 64 scans were recorded with and without phase-cycling, respectively. For ^19^F cw-photo-CIDNP, 16 scans were recorded.

### Computational Methods

Microsolvated models for FMN (3′S_0_)^54^ and tryptophan (8Zpg^+^)^55^ were used as input structures, whereas atoms were modified using Avogadro (Avogadro Version 1.0.2) to yield the respective fluorinated derivatives.^56^ Geometry optimization was performed with the B3LYP functional in combination with the def2-TZVP basis set.^57^ Hyperfine coupling constants were calculated using the B3LYP functional^58,59^ in conjunction with the IGLO-III basis set.^60^ In all calculations, def2/J was chosen as an auxiliary basis.^61^ An atom-pairwise dispersion correction was applied to account for dispersion forces,^62,63^ and the COSMO approach was used to account for solvation effects.^64^

Photo-CIDNP kinetics were simulated using a MATLAB (version 24.2, MathWorks Inc., Natick, MA, USA) script employing the trust-region-reflective algorithm (implemented via the lsqcurvefit function from the Optimization Toolbox, version 24.2) and the built-in ode45 solver for numerical integration. S/N ratios were determined using the “sinocal” AU program built into TopSpin (version 3.6.5). For the F6 resonance of 6-fluorotryptophan, the noise region was set to −115 ppm to −120 ppm, and the signal region to −121.29 ppm to −122.29 ppm.

## Results

### Hyperfine Mapping of Fluorinated Tryptophan Radicals

For the large magnetic field strengths used (7.05 and 14.10 T), coherent spin mixing is only feasible between the S and T_0_ states of the SCRP, since a large, magnetic-field dependent energetic separation of the T_±_ states from the S and T_0_ states prevents them from mixing with these states.^65^ Under such conditions, the nuclear spin-dependent singlet–triplet mixing (STM) critically depends on the nuclear spin state of a given nucleus:^66^

1 $$\begin{array}{ll} \omega_{ST_{0}}= & \frac{1}{2\hbar }\Delta g_{\mathrm{iso}}\mu_{B}B_{0}\\ & +\frac{1}{2}\underset{i}{\sum }A_{\mathrm{iso},i}m_{i}-\frac{1}{2}\underset{j}{\sum }A_{\mathrm{iso},j}m_{j} \end{array}$$

Besides the Bohr magneton μ_B_, the STM frequency $\omega_{ST_{0}}$ depends on the difference of the *g*_iso_-values, Δ*g*_iso_, of the two radicals forming the SCRP, the applied magnetic field strength *B*_0_, the magnetic nuclear spin quantum number *m* and the isotropic hyperfine coupling constant *A*_iso_ of the *i*-th and *j*-th nuclei in the two radicals, respectively. Since recombination is preferred from one electron spin configuration of the SCRP (in this case, the singlet SCRP), one nuclear spin state accumulates in the recombination product, while the other accumulates in the escape product, i.e., radicals that did not recombine, but instead diffuse apart.^17^ Hence, the escaped radicals bear nuclear hyperpolarization of equal size, but opposite sign compared to the geminate polarization, i.e., the hyperpolarization of the recombination product. However, this hyperpolarization is partially lost during the lifetime of the radicals due to the strongly enhanced nuclear relaxation in the vicinity of the unpaired electron spin.^36^ Consequently, the hyperpolarization of the recombination product is observed. The photo-CIDNP signal intensity immediately following the laser pulse (geminate photo-CIDNP) therefore stems from the recombination polarization of the geminate SCRPs (G-pairs). The term “G-pairs” denotes SCRPs that form through electron transfer from the electron donor to the excited state of the dye molecule. In the high magnetic field limit, the signal intensity stemming from G-pairs is proportional to the isotropic hyperfine coupling constant of the respective nucleus.^36^ Photo-CIDNP experiments using a nanosecond-pulsed laser as irradiation source (tr-photo-CIDNP) were therefore used to extract information about the electronic structure and microsecond kinetics of the radicals generated in the photoreaction of fluorinated tryptophan derivatives with 8-fluoro-7,8-didemethyl-FMN.

For reliable correlation of photo-CIDNP intensities to isotropic hyperfine couplings predicted by other methods, such as EPR or quantum-chemical computations, it is mandatory that moiety-specific cancellation processes during the 2.5 μs sampling pulse duration (such as degenerate electron exchange (DEE)) can be excluded.^67^ Therefore, geminate ^1^H photo-CIDNP was performed first. In the absence of moiety-specific cancellation, a plot of the photo-CIDNP signal intensities of all ^1^H nuclei in the geminate SCRP against the isotropic hyperfine coupling constants obtained from an additional source (in this case, DFT) yields one single linear regression, regardless of the radical to which they belong. Furthermore, analysis of geminate ^1^H photo-CIDNP spectra provides valuable information on the protonation state of the fluorinated tryptophan radical in the G-pair, as some hyperfine coupling constants depend strongly on the presence or absence of an additional proton. The occurrence of the anionic 8-fluoro-7,8-didemethyl-FMN radical and the cationic 6-fluorotryptophan radical have already been confirmed in G-pairs at neutral pH.^52^ For all other fluorinated tryptophan derivatives, however, the protonation state of the radical in the G-pair is hitherto unknown.

Figure 2 shows the dark ^1^H NMR and geminate ^1^H photo-CIDNP spectra for mixtures of 8-fluoro-7,8-didemethyl-FMN and 4-, 5-, 6- or 7-fluorotryptophan. In all cases, the photo-CIDNP signals of H6 and H9 from 8-fluoro-7,8-didemethyl-FMN exhibit emissive and enhanced absorptive enhancement, respectively. A small positive enhancement is also observed for H7, but it is largely obscured by noise.

**Figure 2:**
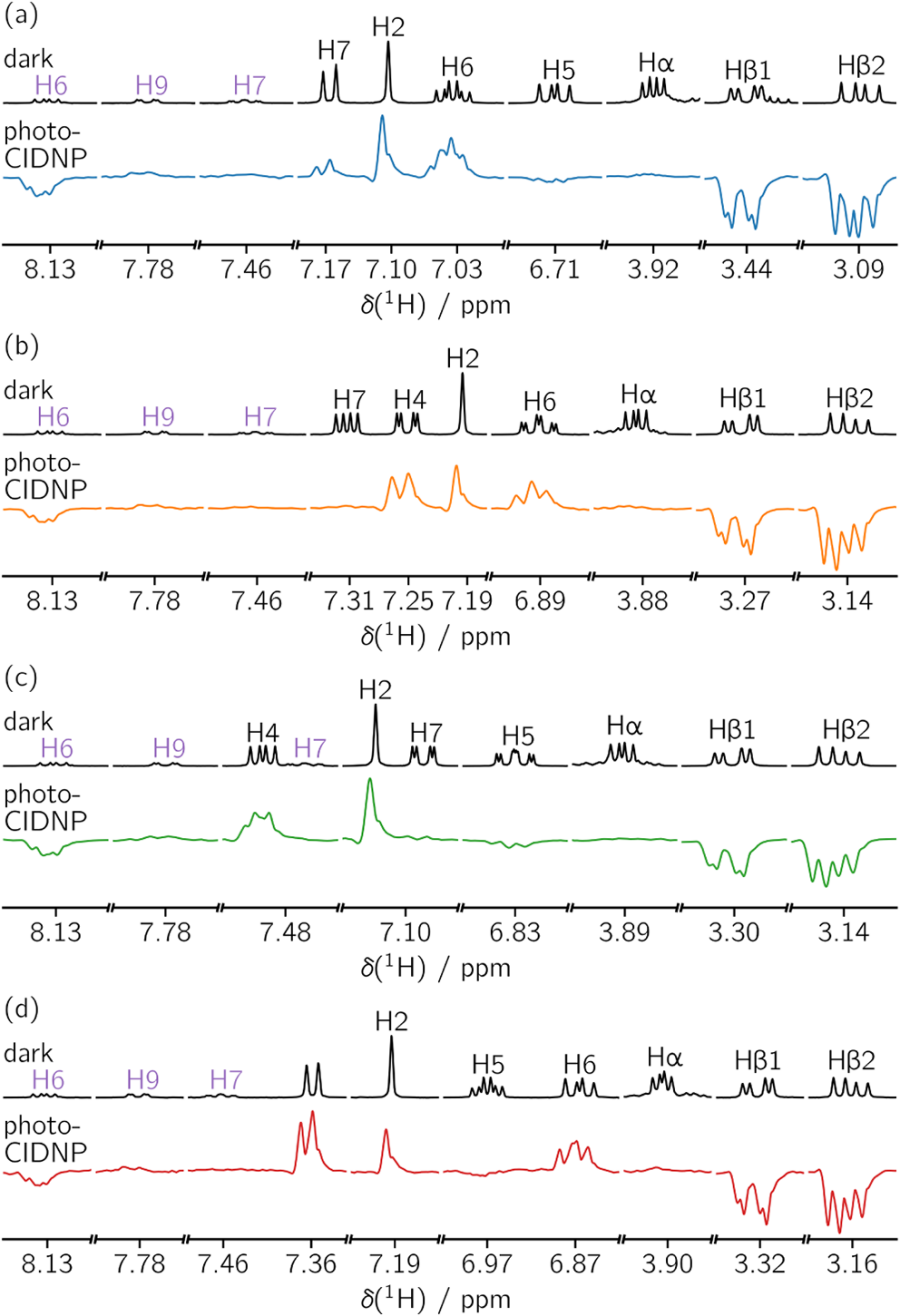
Dark ^1^H NMR (upper) and geminate ^1^H photo-CIDNP (lower) spectra of samples containing 8-fluoro-7,8-didemethyl-FMN and (a) 4-fluorotryptophan, (b) 5-fluorotryptophan, (c) 6-fluorotryptophan, and (d) 7-fluorotryptophan. For easier distinction, the labels of resonances assigned to aromatic protons from 8-fluoro-7,8-didemethyl-FMN are shown in purple.

The sign of the polarization of a specific nucleus *i* (Γ_*i*_, where “+” indicates enhanced absorption and “–” emission) can be determined using Kaptein’s sign rule:^68^

2 $$\Gamma_{i}=\mu \times \varepsilon \times \mathrm{sgn}\left(\Delta g_{\mathrm{iso}}\right)\times \mathrm{sgn}\left(A_{\mathrm{iso},i}\right)$$

μ depends on the spin multiplicity of the excited flavin precursor and is “–” for singlet-born SCRPs and “+” for triplet-born SCRPs. Due to the short lifetime of the flavin’s excited singlet state, G-pairs are typically generated from the triplet state in diffusion-controlled (liquid-state) photo-CIDNP experiments.^1^ *ε* depends on the exit channel of the SCRP; it is “+” for recombination products and “–” for escape products. In the case of G-pairs, the polarization of recombination products is observed. The sign of Δ*g*_iso_ = *g*_iso,1_ – *g*_iso,2_ depends on the isotropic *g*-values of the radicals forming the SCRP. Within one radical, the sign of the isotropic hyperfine coupling constant *A*_iso,*i*_ of the nucleus *i* then determines the sign of the polarization.

Since the signs of the ^1^H resonances from aromatic protons in 8-fluoro-7,8-didemethyl-FMN do not change in solutions with different fluorinated tryptophan derivatives, it can be concluded that the sign of Δ*g*_iso_ does not change among the examined fluorinated tryptophans. With DFT-predicted isotropic hyperfine coupling constants of −9.37 and 2.34 MHz for H6 and H9 of the anionic 8-fluoro-7,8-didemethyl-FMN radical (Table S1), respectively, Δ*g*_iso_ is confirmed to be positive for the anionic 8-fluoro-7,8-didemethyl-FMN radical in all cases. Hence, the isotropic *g*-values of all studied fluorinated tryptophan radicals must be lower than that of the anionic 8-fluoro-7,8-didemethyl-FMN radical. The same holds for the FMN-tryptophan pair, where the isotropic *g*-value of the cationic tryptophan radical is lower than that of the anionic flavin radical (TrpH^•+^: *g*_iso_ = 2.00226–2.00325,^69^ and FMN^•–^: *g*_iso_ = 2.0036).^70^

A comparison of the geminate ^1^H photo-CIDNP intensities from aromatic protons and the DFT-predicted isotropic hyperfine coupling constants of the fluorinated tryptophan derivatives (Table S2) suggests that the electronic structure of the indole moiety does not change significantly depending on the fluorination position. In all cases, H5 exhibits a (weak) emissive enhancement. H4 exhibits the strongest absorptive enhancement, followed by H6 and H2. The H7 resonance is only weakly polarized. Larger errors are expected in cases of overlapping signals when evaluating the photo-CIDNP intensities by integration, especially for the weaker of the two overlapping resonances. Such overlap occurs between H2 and H7 in 6-fluorotryptophan, as well as between H4 of 6-fluorotryptophan and H7 of 8-fluoro-7,8-didemethyl-FMN. The aliphatic protons Hβ1 and Hβ2 exhibit the strongest enhancement, which is emissive in all cases, while Hα exhibits a weak absorptive enhancement. These results align with those obtained for the parent molecule tryptophan when using FMN as photosensitizer. In this case, strong enhancement is observed for Hβ1, Hβ2, H2, H4 and H6, while weak enhancement is observed for Hα, H5 and H7.^10^

Interestingly, regarding aromatic protons, DFT predicts the strongest variation between the isotropic hyperfine coupling constants of the various fluorinated tryptophan radicals for proton H2 that is not attached to the benzene ring. Its hyperfine coupling is approximately 5–6 MHz stronger in 6-fluorotryptophan than in the other fluorinated tryptophan derivatives. Although the geminate ^1^H photo-CIDNP intensity is equal for H2 and H4, the predicted isotropic hyperfine coupling constant is slightly larger for H2.

The gathered information was evaluated by plotting geminate ^1^H photo-CIDNP intensities against DFT-predicted hyperfine coupling constants (Figures 3 and S3) for all aromatic protons. The aliphatic protons (Hβ1, Hβ2 and Hα) were excluded for this analysis, since their hyperfine coupling depends on the dihedral angle between the Cβ–Hβ bond and the 2p_*z*_ orbital at C3, projected onto the plane perpendicular to the C3–Cβ bond.^71^ Therefore, they are not easily predicted by DFT, since only one optimized geometry is used as the input structure for the prediction of electronic parameters (hyperfine couplings).

**Figure 3:**
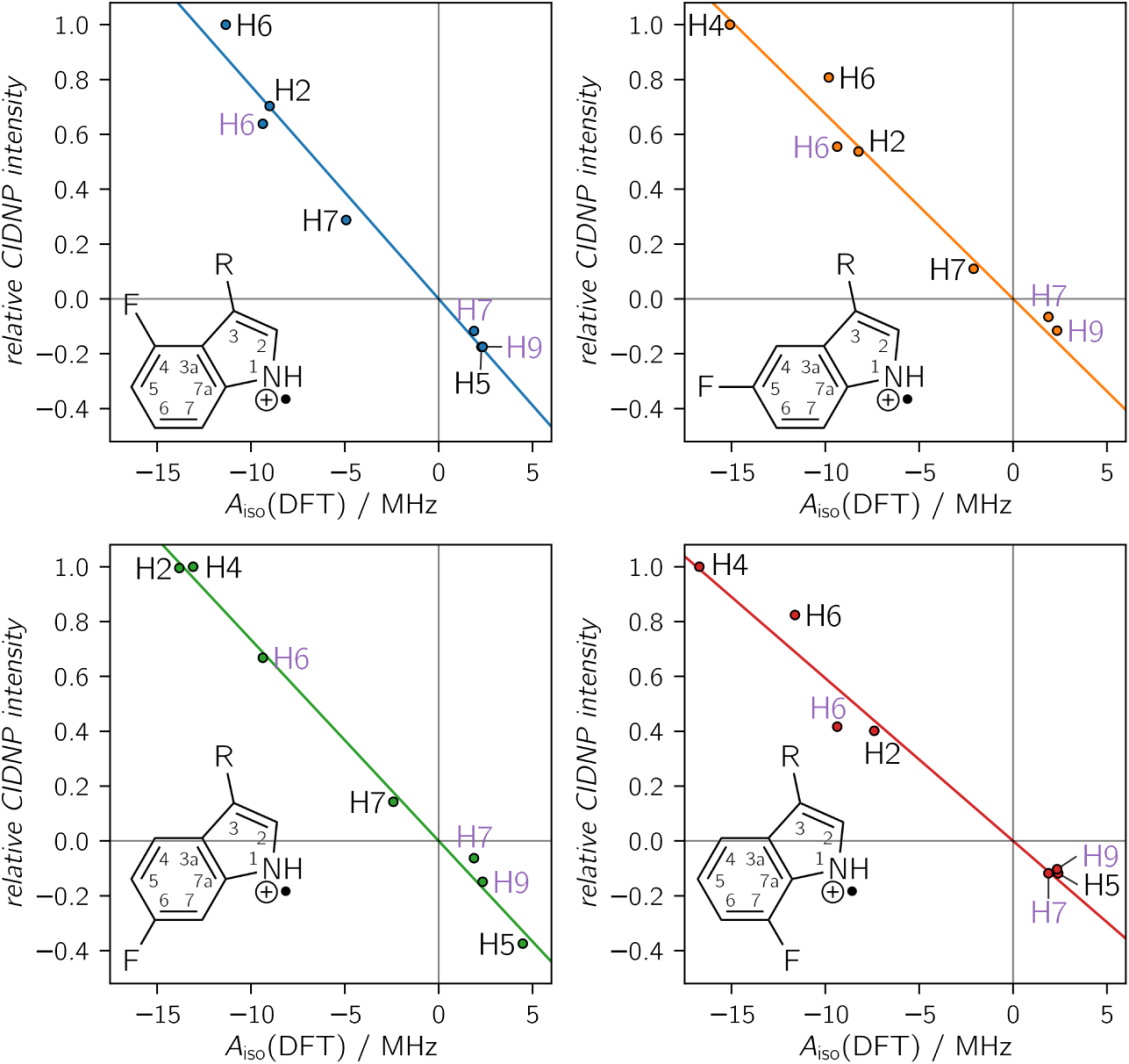
Geminate ^1^H photo-CIDNP/DFT correlation plots using DFT-predicted isotropic hyperfine coupling constants for the cationic radicals of 4-fluorotryptophan (upper left, *m* = −0.0779 MHz^–1^, *R* ^2^ = 0.9769), 5-fluorotryptophan (upper right, *m* = −0.0675 MHz^–1^, *R* ^2^ = 0.9699), 6-fluorotryptophan (lower left, *m* = −0.0734 MHz^–1^, *R* ^2^ = 0.9939) and 7-fluorotryptophan (lower right, *m* = −0.0593 MHz^–1^, *R* ^2^ = 0.9684). The solid lines represent linear regression fits based on the available data points, constrained to go through the origin. Geminate ^1^H photo-CIDNP intensities were extracted from the spectra shown in Figure 2. The intensities were multiplied by –1 for protons in 8-fluoro-7,8-didemethyl-FMN to account for Kaptein’s sign rule.^68^ The labels assigning these resonances are shown in purple for easier distinction. The isotropic hyperfine coupling constants obtained from DFT and observed photo-CIDNP intensities are listed in Tables S1, S2 and S3.

From the correlation plots, two conclusions can be drawn: First, the correlation is stronger for all fluorinated tryptophan derivatives when using DFT-predicted hyperfine coupling constants of the cationic radical rather than the neutral radical. This confirms the presence of the cationic tryptophan radical in the G-pair in all cases. Second, the geminate ^1^H photo-CIDNP intensities of all ^1^H nuclei of 8-fluoro-7,8-didemethyl-FMN and the respective fluorinated tryptophan derivative can be adequately described by a single linear regression. Hence, moiety-specific cancellation can be safely excluded for all examined fluorinated tryptophan derivatives under the chosen experimental conditions (neutral pH and a 2.5 μs sampling pulse). This can be rationalized by different protonation states of the radical in the G-pair and tryptophan in bulk solution, as is the case for tryptophan at neutral pH.^10^ Deprotonation of the radical after the G-pair lifetime (<10 ns) renders DEE with the protonated, ground-state fluorinated tryptophan derivatives unfeasible. Therefore, cancellation specific to the fluorinated tryptophan derivatives is not observed. The microsecond photo-CIDNP kinetics will be examined in more detail below.

The correlations established between the relative photo-CIDNP signal intensities and the DFT-predicted isotropic hyperfine coupling constants can be used to calculate the isotropic hyperfine coupling constants of the aliphatic protons Hβ1, Hβ2, and Hα. This is achieved by dividing the photo-CIDNP signal intensity of a given aliphatic proton (see Table S3) by the slope of the respective correlation plot (see Figure 3). As previously mentioned, these isotropic hyperfine coupling constants are difficult to predict using DFT, as the geometry of these protons in relation to the aromatic ring is flexible. The values obtained range from 22.6 to 26.1 MHz for Hβ1 (the downfield-shifted Hβ proton), from 27.5 to 34.1 MHz for Hβ2 (the upfield-shifted Hβ proton), and from −0.8 MHz to −1.4 MHz for Hα.

Geminate ^19^F photo-CIDNP can be used to probe the isotropic hyperfine coupling constants of ^19^F nuclei.^52^ Because the method relies on comparing signal intensities, at least two nuclei are required in the SCRP. To fulfill this condition, a monofluorinated flavin (8-fluoro-7,8-didemethyl-FMN) was used as photosensitizer. The chemical shift of all fluorinated tryptophan derivatives is between −120 ppm and −140 ppm, and strong (emissive) enhancements are observed for the ^19^F nuclei in 4- and 6-fluorotryptophan (Figure 4). This is expected since these are the benzene ring positions where strong ^1^H photo-CIDNP enhancements were observed in the fluorinated tryptophan derivatives bearing a proton in the respective positions (see Figure 2). Also, the overall spin distribution does not appear to change significantly in the benzene ring of the indole, as evidenced by the moderate changes in the isotropic hyperfine coupling constants. As weak enhancements were observed for ^1^H nuclei in positions 5 and 7, it is not surprising that ^19^F nuclei in these positions also exhibit weak photo-CIDNP enhancements.

**Figure 4:**
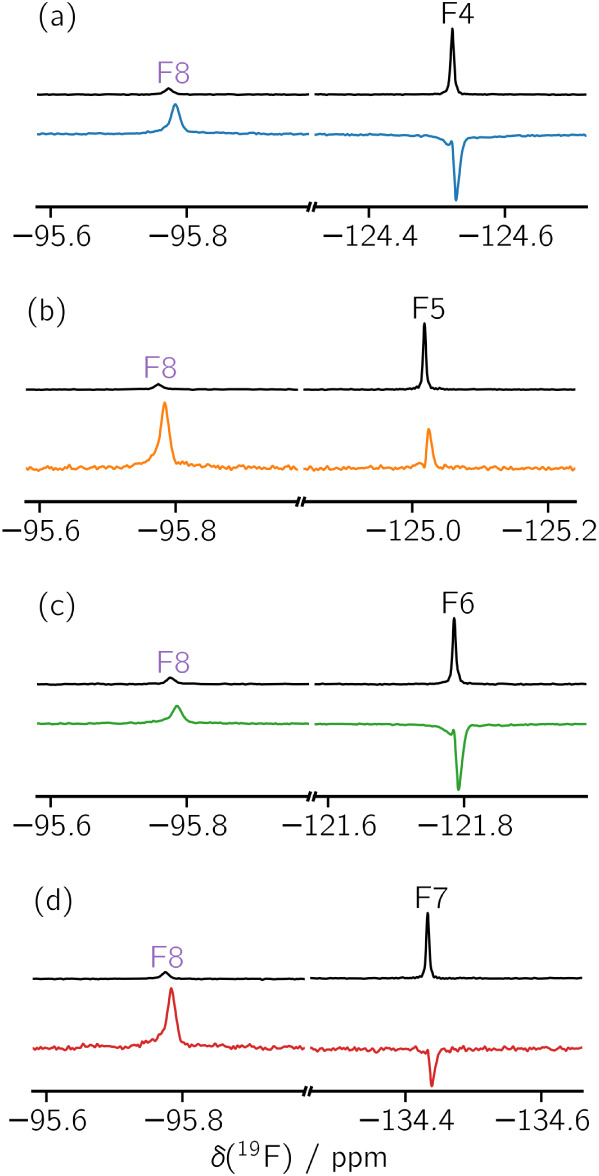
Dark ^19^F NMR (upper) and geminate ^19^F photo-CIDNP (lower) spectra of samples containing 8-fluoro-7,8-didemethyl-FMN and (a) 4-fluorotryptophan, (b) 5-fluorotryptophan, (c) 6-fluorotryptophan, and (d) 7-fluorotryptophan. For easier distinction, the labels assigned to resonances from 8-fluoro-7,8-didemethyl-FMN are shown in purple.

The geminate ^19^F photo-CIDNP signal intensities were correlated with DFT-predicted isotropic hyperfine coupling constants (Figure 5 and Table 1). Previous analyses of geminate ^1^H photo-CIDNP revealed that cationic radicals of the fluorinated tryptophan derivatives are present in G-pairs (see Figure 3). The agreement between DFT-predicted ^19^F hyperfine couplings and geminate ^19^F photo-CIDNP intensities is also excellent for 4-, 5- and 6-fluorotryptophan when hyperfine data of the respective cationic radical species are used (*R* ^2^ > 0.9500). Such good correlations can only be achieved when the ^19^F hyperfine coupling constant of the anionic 8-fluoro-7,8-didemethyl-FMN is also reliably predicted by DFT.

**Figure 5:**
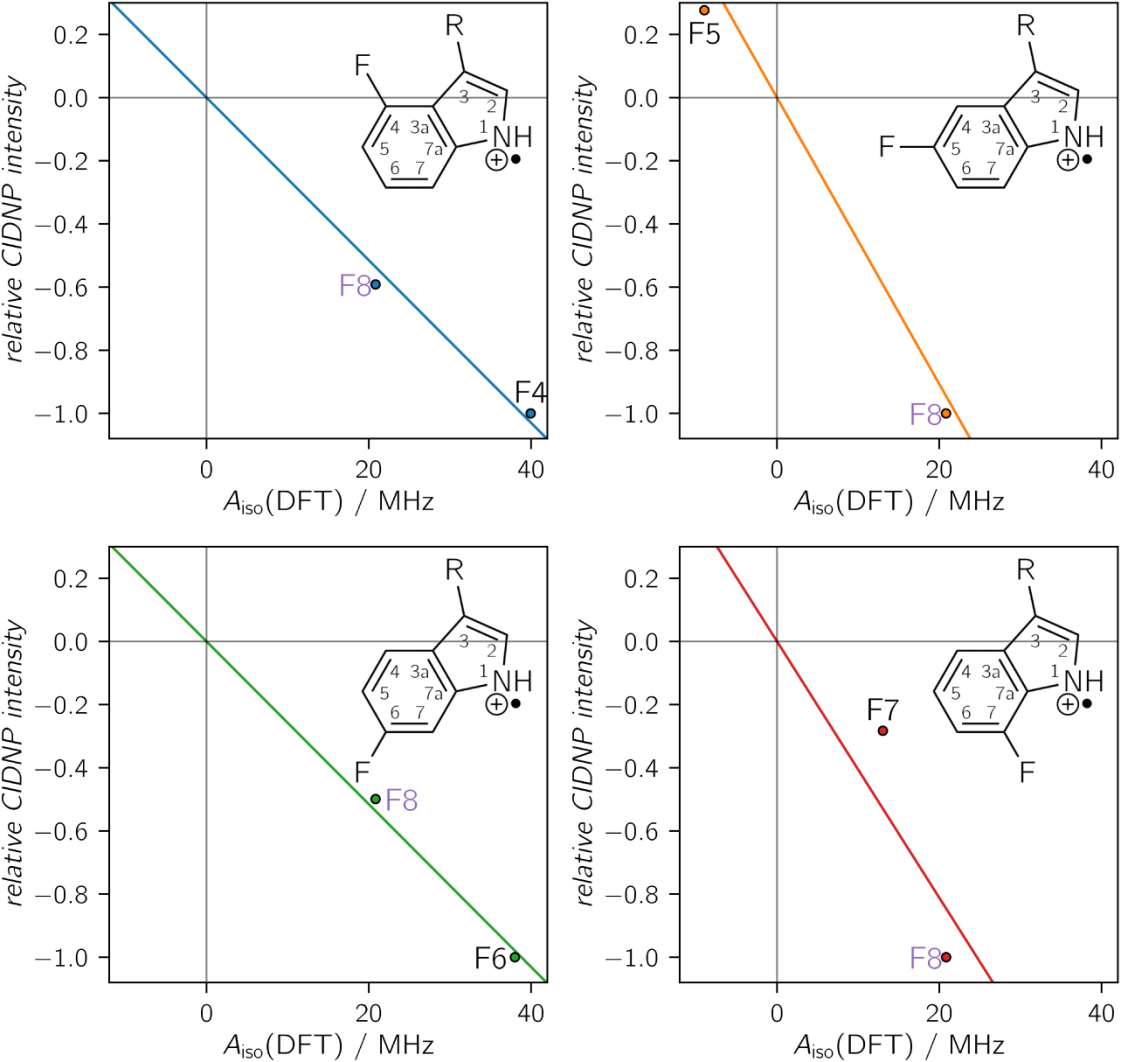
^19^F photo-CIDNP/DFT correlation plots using DFT-predicted isotropic hyperfine coupling constants for the cationic radicals of 4-fluorotryptophan (upper left, *R* ^2^ = 0.9537), 5-fluorotryptophan (upper right, *R* ^2^ = 0.9781), 6-fluorotryptophan (lower left, *R* ^2^ = 0.9851) and 7-fluorotryptophan (lower right, *R* ^2^ = 0.6712). The solid lines represent linear regression fits based on the two available data points, constrained to go through the origin. Photo-CIDNP intensities are listed in Table 1 and were extracted from the spectra shown in Figure 4. For easier distinction, the labels assigned to resonances from 8-fluoro-7,8-didemethyl-FMN are shown in purple.

**Table 1:** Geminate ^19^F Photo-CIDNP Intensities of Samples Containing the Fluorinated Tryptophan Derivatives and 8-Fluoro-7,8-Didemethyl-FMN (Figure 4), and DFT-Predicted Isotropic Hyperfine Coupling Constants of ^19^F Nuclei in the Cationic and Neutral Fluorinated Tryptophan Radicals^a^

	8-Fluoro-7,8-didemethyl-FMN	Fluorinated tryptophan	Cationic radical	Neutral radical
Electron donor	Rel. *A*_iso_ (CIDNP)	Rel. *A*_iso_ (CIDNP)	*A*_iso_/MHz (DFT)	*A*_iso_/MHz (DFT)
4-Fluorotryptophan	–0.59	–1.00	39.96	30.89
5-Fluorotryptophan	–1.00	0.29	–8.94	–5.30
6-Fluorotryptophan	–0.50	–1.00	38.00	29.86
7-Fluorotryptophan	–1.00	–0.28	13.05	5.24

a The intensities were normalized with respect to the stronger signal in each measurement. The intensities of the 8-fluoro-7,8-didemethyl-FMN were multiplied by −1 to account for Kaptein’s sign rule.^68^ An isotropic hyperfine coupling constant of 20.84 MHz was predicted for the anionic 8-fluoro-7,8-didemethyl-FMN radical.

Among the fluorinated tryptophan derivatives, 7-fluorotryptophan exhibits the poorest correlation in geminate ^1^H photo-CIDNP when using DFT-predicted isotropic hyperfine coupling constants from the cationic radical, and the best correlation when using the neutral radical. Possibly, deprotonation is faster in 7-fluorotryptophan than in the other fluorinated tryptophan derivatives. This would lead to a stronger contribution of the neutral radical to the photo-CIDNP polarization. Since the ^19^F nucleus in the neutral radical has a substantially lower predicted isotropic hyperfine coupling constant than the cationic radical (5.24 MHz vs 13.05 MHz), this could explain the lower photo-CIDNP intensities. It is also possible that the isotropic hyperfine coupling constant is overestimated by DFT. Definitive conclusions on this topic cannot be drawn at this time.

### Cw-Photo-CIDNP

To determine which fluorinated tryptophan derivative is best suited for ^19^F cw-photo-CIDNP experiments, an equimolar mixture of all fluorinated tryptophan derivatives was measured using this method (Figure 6a). Both fluorescein and FMN were used as photosensitizers under similar experimental conditions, i.e., same optical density at the irradiation wavelength (445 nm). For both photosensitizers, 5-fluoro- and 7-fluorotryptophan exhibit the weakest cw-photo-CIDNP signals, corresponding to the low isotropic hyperfine coupling constants of the respective fluorine nuclei in the radicals (Table 1). However, the signal intensity from 6-fluorotryptophan is approximately twice that of 4-fluorotryptophan, despite their similar isotropic hyperfine coupling constants. This clearly shows that hyperfine couplings are not the only decisive factor regarding the photo-CIDNP enhancement. In cw-photo-CIDNP, the radicals’ paramagnetic relaxation rates (see below), electronic structure of the radicals (*g*-values and hyperfine couplings; eq 1), electron transfer rates to the excited photosensitizer, diffusion speed, photodegradation rates, and relaxation dynamics of the respective nuclei in the diamagnetic ground state molecules, including cross-relaxation and cross-correlation,^43^ govern the photo-CIDNP pumping rate. From the investigation of geminate ^19^F/^1^H photo-CIDNP and DFT predictions of hyperfine couplings (Tables S2 and S3) and *g*_iso_-values (Table S4), it can be inferred that the electronic structure does not significantly differ between 4- and 6-fluorotryptophan radicals. Considering that the second radical of the SCRP (the FMN radical) is the same for both tryptophan derivatives, this means that the distribution of singlet–triplet mixing frequencies (eq 1) does not differ strongly. The diffusion speed is also not expected to change significantly, since all derivatives have the same molecular weight and similar shape. Photodegradation is observed in consecutive measurements of ^19^F cw-photo-CIDNP spectra (Figure 6b), but it is not strong enough to be relevant in a 16-scan measurement. Additionally, the evolution of the ^19^F cw-photo-CIDNP signal intensity in consecutive measurements is similar for F4 and F6 of 4- and 6-fluorotryptophan, respectively (Figure S4). This suggests that the photodegradation rates are similar for both derivatives, or that the photodegradation of FMN governs the loss of photo-CIDNP polarization over multiple measurements.

**Figure 6:**
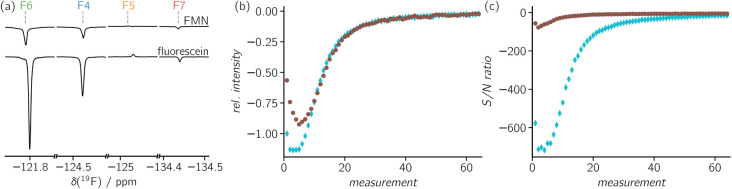
(a) 16-scan ^19^F cw-photo-CIDNP spectra of a sample containing 2 mM of each 4-, 5-, 6-, and 7-fluorotryptophan, as well as either 200 μM FMN or 100 μM fluorescein (both with an OD_445_ nm of 2.5). The noise level is equal in both spectra. Intensity ratios F6:F4:F5:F7 are −1:–0.64:0.03:–0.04 (FMN) and −1:–0.46:0.02:–0.04 (fluorescein). (b) Signals of F6 from 6-fluorotryptophan in the same samples. Dark ^19^F NMR spectra (128 scans, positive amplitudes) were acquired before and after 64 consecutive 16-scan ^19^F cw-photo-CIDNP measurements. The displayed ^19^F cw-photo-CIDNP spectra (16 scans, negative amplitudes) are the third and tenth measurement of the 64 measurements. (c) Evolution of relative signal intensities (integrals) and S/N ratios of the F6 signal in the consecutive ^19^F cw-photo-CIDNP measurements with FMN (brown circles) and fluorescein (cyan diamonds).

However, longitudinal relaxation rates were found to be different for the fluorine atoms in the fluorinated tryptophan derivatives. Of all the investigated fluorinated tryptophan derivatives, the highest longitudinal relaxation rate measured at slightly higher temperature (298 K) and higher magnetic field (14.10 T) was found to be 0.99 s^–1^ for 4-fluorotryptophan, while the lowest relaxation rate of 0.67 s^–1^ was found for 6-fluorotryptophan.^72^ Longitudinal relaxation already occurs during sample irradiation (0.5 s) and counteracts the buildup of photo-CIDNP polarization, thus explaining the stronger photo-CIDNP enhancement observed in 6-fluorotryptophan as compared to 4-fluorotryptophan.

In a sample containing 1 mM 6-fluorotryptophan and 200 μM FMN, the enhancement factor between dark ^19^F NMR and ^19^F photo-CIDNP NMR (both 16 scans) was determined to be −16.5 (7.05 T, with 300 mW irradiation at 445 nm for 0.5 s). Using a 32-scans cw-photo-CIDNP spectrum, which is the better comparison given that our ^19^F cw-photo-CIDNP experiments employed destructive phase cycling,^20^ the enhancement factor is −36. This is comparable to the 40-fold enhancement observed for 4 mM 3-fluorotyrosine with 200 μM FMN (14.10 T, with 25 W irradiation at 448 and 512 nm).^43^ An even stronger enhancement is expected for lower concentrations of 6-fluorotryptophan, considering that the dark NMR signal grows linearly with concentration, while the photo-CIDNP intensity reaches a maximum (approximately 1 mM for tryptophan when using 500 μM FMN with a 0.1 s irradiation time).^17^

Regarding the choice of photosensitizer, fluorescein provides an S/N ratio approximately ten times higher for multiple irradiation cycles (see Figure 6c). Initially, the signal intensity (integral) is only about twice as high for fluorescein as compared to FMN. After only ten 16-scan photo-CIDNP acquisitions, the signal intensity is similar for both (see Figure 6b). This discrepancy can be explained by a broadening of the ^19^F resonances observed for all examined fluorinated tryptophan derivatives when using FMN as the photosensitizer (Figure S5). Interestingly, only slight line broadening was observed for 2- and 3-fluorotyrosine when using either FMN or fluorescein as photosensitizer (Figure S6). We conclude that an irreversible photooxidation of the fluorinated tryptophan derivatives causes this behavior. Additional remarks and conclusions regarding this process can be found in the Supporting Information.

### Microsecond Photo-CIDNP Kinetics of 6-Fluorotryptophan

After the generation of G-pairs, which recombine on a short nanosecond time scale, the photo-CIDNP intensity changes on a microsecond time scale.^11,73^ The evolution of photo-CIDNP polarization in freely diffusing SCRPs is usually dominated by three processes: (1) the generation of so-called free radical pairs (F-pairs)^74^ from radicals that escaped geminate recombination, which in turn generate photo-CIDNP hyperpolarization, (2) unselective radical recombination reactions that transfer the “unfavored” spin state accumulated in the escape products back to the diamagnetic ground state, which results in cancellation of the photo-CIDNP intensity, and (3) *T*_1_ relaxation in the radicals, which counteracts cancellation of the photo-CIDNP polarization from process (2).^36^ If *T*_1_ relaxation is sufficiently fast, cancellation of the photo-CIDNP intensity due to unselective recombination does not occur, as was observed for the fluorine nucleus in 4-fluorophenol.^75^ A fourth mechanism that quickly cancels nuclear hyperpolarization can be observed under specific conditions: when the protonation pattern of the diamagnetic ground state molecule and the thermodynamically favored protonation state of the respective radical are identical, degenerate electron exchange (DEE) transforms the “unfavored” nuclear spin of the escape products back to the diamagnetic ground state.^67^ The presence of this cancellation mechanism depends mainly on the pH of the solution, and occurs only when the radical concentration is lower than the concentration of the diamagnetic ground state molecule. For tryptophan, DEE is observed below the p*K*_a_ of the tryptophanyl radical (p*K*_a_ = 4.3)^76^ and is therefore irrelevant under physiological conditions.^6^

The time evolution of photo-CIDNP polarization can be described using a set of differential equations proposed by Vollenweider and Fischer:^11,73^

3 $$R\left(t\right)=\frac{R_{0}}{1+k_{t}R_{0}t}$$

4 $$\begin{array}{ll} \frac{dP_{R}}{dt}= & -k_{t}P_{R}R\left(t\right)-k_{t}\beta R{\left(t\right)}^{2}\\ & -\frac{P_{R}}{T_{1}}-k_{\mathrm{ex}}CP_{R} \end{array}$$

5 $$\frac{dP_{P}}{dt}=k_{t}P_{R}R\left(t\right)+k_{t}\beta R{\left(t\right)}^{2}+k_{\mathrm{ex}}CP_{R}$$

Here, *R*(*t*) is the time-dependent radical concentration, *R*_0_ denotes the initial radical concentration, *k*_t_ is the radical termination rate constant and *k*_ex_ the DEE rate constant, *C* is the concentration of diamagnetic ground state molecules involved in DEE, *T*_1_ is the nuclear paramagnetic relaxation time of the observed nucleus, β represents the polarization per F-pair, and *P*_R_ and *P*_P_ are the polarizations of radicals and diamagnetic ground state molecules (products), respectively.

To demonstrate the influence of large hyperfine anisotropy, and consequently, significant *T*_1_ relaxation in fluorine nuclei of radicals on microsecond photo-CIDNP kinetics, tr-photo-CIDNP measurements were carried out with various delays between pulsed laser excitation and the sampling pulse (Figure 7). The sampling pulse duration was set to 1 μs to increase the resolution. FMN was used as photosensitizer because fluorescein did not produce an appreciable signal intensity, presumably due to its low intersystem crossing rate.^13^ A fresh sample was used for each measurement to avoid line broadening and photobleaching. Strong hyperfine anisotropies of 218 and 217 MHz (cationic)/163 and 157 MHz (neutral) were predicted for the 4-fluorotryptophan and 6-fluorotryptophan radicals, respectively (Table S5). 6-Fluorotryptophan was chosen for these measurements since it exhibited the strongest ^19^F photo-CIDNP enhancement under continuous-wave irradiation. Ideally, measurements would be carried out at equal magnetic field strengths to achieve the best comparability of paramagnetic relaxation times between ^1^H and ^19^F. However, ^1^H measurements were conducted at 14.10 T and ^19^F measurements at 7.05 T because the comparably long ^19^F 90° pulse duration on our spectrometer operating at 14.10 T (∼21 μs) compared to the short ^19^F 90° pulse duration on the spectrometer operating at 7.05 T (∼7.7 μs) would lead to a weak signal intensity for the 14.10 T spectrometer when using a sampling pulse duration of 1 μs. Since a TXI probe optimized for ^1^H observation was only available on the spectrometer operating at 14.10 T, we chose it for the ^1^H measurements.

**Figure 7:**
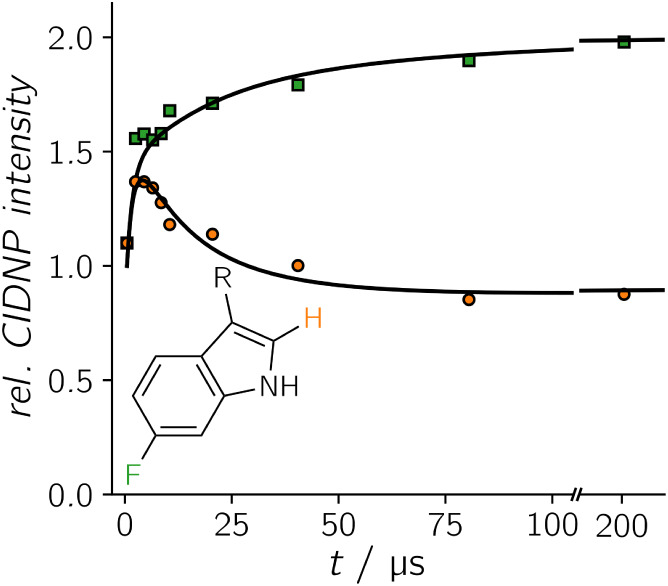
Microsecond kinetics of photo-CIDNP polarization for H2 (orange circles) and F6 (green squares) of 6-fluorotryptophan in the photoreaction with FMN. For a better comparability, the absolute values of the photo-CIDNP signal intensities were evaluated. The parameters for the simulation (solid lines) were: *R*_0_ *k*_t_: 2.8 × 10^5^ s^–1^ (both), *T*_1_: 8 μs (F6) and 55 μs (H2). *P* ^G^ was set to 1, *k*_ex_ was set to 0 and γ was set to 2.8. The inset shows 6-fluorotryptophan and the nuclei whose signals were evaluated, H2 (orange) and F6 (green). The spectra from which signal intensities were taken from are depicted in Figure S8.

The obtained data were fitted to the model proposed by Vollenweider and Fischer (eqs 3–5).^73^ The DEE rate constant *k*_ex_ was set to zero since the presence of DEE results in the rapid cancellation of photo-CIDNP polarization, which was not observed in our case (see above). For the parent molecule tryptophan, fast deprotonation (*k*_dep_ = 1.5 × 10^6^ s^–1^)^76^ of the cationic tryptophan radical has been shown to inhibit DEE on the microsecond time scale.^11^ However, even though deprotonation is fast, the DEE rate is comparable: for the tryptophan derivative *N*-acetyl tryptophan, the DEE rate constant was found to be *k*_ex_ = 9 × 10^8^ M^–1^ s^–1^.^11^ Hence, in a pH-neutral solution containing 1 mM *N*-acetyl tryptophan, the expected photo-CIDNP decay rate by DEE is 9 × 10^5^ s^–1^. Assuming that the deprotonation and DEE rates are similar for the cationic 6-fluorotryptophan radical, DEE will only influence the photo-CIDNP kinetics within the first few hundred nanoseconds. In principle, it is possible to extend the differential equations to account for this behavior. However, it is prudent to not overparameterize the problem. As the time resolution of this method is in the short microsecond range, the rate constant *k*_ex_ was set to zero, given that DEE is only expected to occur within the first few hundred nanoseconds.

Another issue not addressed in the treatment proposed by Vollenweider and Fischer is the change in photo-CIDNP generation due to deprotonation. In this study, G-pairs consist of anionic flavin radical^3,4^ and cationic fluorinated tryptophan radical (see above). However, the deprotonated fluorinated tryptophan radical occurs in F-pairs, which has a different spin distribution, and therefore different hyperfine coupling constants. This affects the STM frequency distribution, and consequently, the polarization generation by F-pairs. Most importantly, the change in the isotropic hyperfine coupling constant of a given nucleus affects its photo-CIDNP generation since the isotropic hyperfine coupling constant is the weighting factor in the STM spin-sorting process (eq 1). This issue can be adequately addressed by applying a scaling factor to all experimentally obtained photo-CIDNP intensities. This allows for deviations from *P*(0) = 1. In addition, to account for the finite duration of the RF detection pulse, the effective time axis was corrected by adding half of the pulse duration (0.5 μs) to the nominal laser–detection delay. The best fits were obtained with scaling factors of 1.1 in both cases.

Simulating the kinetics of H2 yielded *k*_t_ *R*_0_ = 2.8 × 10^5^ s^–1^ and *T*_1_ = 55 μs. Assuming that no double excitation of FMN occurs during the 4–7 ns laser pulse, the maximum possible initial concentration of radicals *R*_0_ would be 7.5 × 10^–5^ M for a sample of 2 × 10^–4^ M FMN, using a triplet quantum yield of 0.375 ^77^ and considering that SCRP formation occurs from the triplet state.^1^ In this case, the minimal radical termination rate constant *k*_t_ would be 3.7 × 10^9^ M^–1^ s^–1^, which is large compared to the *k*_t_ of 2 × 10^9^ M^–1^ s^–1^ for the photoreaction of 2,2′-dipyridyl with tryptophan under comparable conditions and with similar concentrations.^11^ Since the comparably long nuclear relaxation time permits observation of polarization transfer from the escaped radicals to the diamagnetic ground state (product), which is described by the first term of eqs 4 and 5, the results from H2 are expected to be more reliable regarding *k*_t_ *R*_0_ than those from F6, where the photo-CIDNP kinetics are primarily dominated by the fast paramagnetic nuclear relaxation.

For the simulation of the F6 photo-CIDNP kinetics, *k*_t_ *R*_0_ was fixed to the value obtained for H2, since the samples were measured under equal conditions. The only difference were the magnetic field strengths (7.05 T for F6 vs 14.10 T for H2). Although the STM frequency depends on the magnetic field strength (eq 1), the overall radical termination rate, which includes radical termination without F-pair formation, should not change significantly enough to justify using different *k*_t_ *R*_0_ values for H2 and F6.

The paramagnetic nuclear relaxation time *T*_1_ of 8 μs obtained from the photo-CIDNP kinetics of F6 is very short compared to the one obtained for H2 (55 μs). This difference can explain the different microsecond kinetics for protons and fluorine nuclei. Due to the fast paramagnetic nuclear relaxation, less cancellation of product polarization from escaped radicals is expected, and hence, the steady-state polarization is higher for F6 than for H2. As the cw-photo-CIDNP enhancement heavily depends on the microsecond steady-state polarization, fluorine nuclei with their short paramagnetic nuclear relaxation times are of particular interest when a high cw-photo-CIDNP polarization is desired.

## Discussion

As revealed by ^1^H tr-photo-CIDNP, all four of the studied fluorinated tryptophan derivatives form the cationic tryptophan radical in the G-pair. Utilizing 8-fluoro-7,8-didemethyl-FMN as a reference, the ^19^F nuclei in cationic radicals of 4-fluorotryptophan and 6-fluorotryptophan were shown to have the strongest isotropic hyperfine coupling constants, with DFT-predicted values of 39.96 and 38.00 MHz, respectively. While the isotropic hyperfine coupling constant is crucial for photo-CIDNP intensity, the longitudinal relaxation time strongly influences the photo-CIDNP intensity under cw irradiation. The different longitudinal relaxation rates of the ground-state molecules resulted in twice the cw-photo-CIDNP polarization in 6-fluorotryptophan as compared to 4-fluorotryptophan under the chosen conditions. A ^19^F photo-CIDNP enhancement of −36 was determined when using 1 mM 6-fluorotryptophan and 200 μM FMN as photosensitizer. In the case of fluorinated tryptophan derivatives, fluorescein was determined to be the superior photosensitizer, since the photochemical reaction between FMN and fluorinated tryptophan seems to involve a fluorinated species with a similar structure, but a different transverse relaxation time.

The short paramagnetic *T*_1_ relaxation time (8 μs) obtained for F6 of the 6-fluorotryptophan radical indicates a high hyperfine anisotropy for this fluorine nucleus. DFT calculations support this conclusion as well (Table S5), predicting a large anisotropy of 218.1 and 163.4 MHz for the cationic and neutral radical of 6-fluorotryptophan, respectively. A large anisotropy of 216.8 and 156.7 MHz is also predicted for the cationic and neutral radical of 4-fluorotryptophan, respectively. Photo-CIDNP can not only be observed when SCRP formation and recombination are diffusion-controlled, but also when the redox-active moieties have a fixed distance and orientation. Examples include photosynthetic reaction centers, in which photo-CIDNP polarization can be observed in magic angle spinning (MAS) NMR,^27,29^ and cysteine-devoid LOV domains, in which SCRPs form between a protein-bound flavin and a tryptophan residue.^25,26^ LOV domains are an extensively studied model for photo-CIDNP in proteins.^33,78,79^ Experiments including fluorine nuclei in the redox-active moieties, including fluorinated flavin derivatives,^52^ may help to develop a more complete understanding of the dynamics and interplay of the different solid-state mechanisms involved, and can provide more data for investigating the field-dependency of solid-state photo-CIDNP.^80^ As in those cases, the hyperfine anisotropy is crucial for the generation of photo-CIDNP hyperpolarization,^80,81^ the obvious choices are 4-fluorotryptophan and 6-fluorotryptophan.

The incorporation of unnatural amino acids into proteins is a widely used technique among spectroscopists.^47^ However, site-specific incorporation is not feasible by ^13^C enrichment, since the expression system cannot distinguish between the chemically equivalent enriched and nonenriched molecules. 4-, 5- and 6-fluorotryptophan can be incorporated into proteins by replacing all tryptophan residues with their fluorinated analogues simultaneously using selective pressure incorporation,^82^ while 7-fluorotryptophan can be incorporated site-specifically using amber codon suppression.^83^ Therefore, 7-fluorotryptophan can be used when site-specific incorporation is necessary. For example, it can be used to identify the residue responsible for SCRP formation when the protein backbone contains multiple tryptophan residues. Unfortunately, protocols for the site-specific incorporation of either 4-fluorotryptophan or 6-fluorotryptophan do not yet exist. However, even though the cationic 7-fluorotryptophan radical has a lower predicted hyperfine anisotropy (80.84 MHz), it is still high compared to the hyperfine anisotropies of ^13^C nuclei in the cationic tryptophan radical.^84^ Considering that the highest anisotropy of 80.9 MHz is predicted for C3, which is already observable in natural abundance photo-CIDNP measurements,^26^ even 7-fluorotryptophan is expected to produce significant photo-CIDNP enhancements.

When transitioning from fluorinated amino acids in solution to surface-exposed amino acids in proteins, the effect of the large CSA of the ^19^F nucleus should be considered. CSA relaxation becomes significant in proteins with molecular weights over 20 kDa and magnetic field strengths above 5 T,^85^ so broad lines are expected. 4-Fluorotryptophan may also be a valid choice in such cases as the transverse relaxation is then mainly governed by CSA instead of dipole–dipole interactions. Thus, cw-photo-CIDNP polarization may not depend on the differences in relaxation times observed for the free amino acids in solution.^72^ Additionally, intricate changes of the photo-CIDNP intensity in ^19^F nuclei can be expected, which depend on the specific case and are difficult to predict. Depending on the rotational correlation time of the fluorinated amino acid, the strength of the anisotropy and the associated nuclear spin relaxation time, signal inversions of the geminate photo-CIDNP hyperpolarization were observed.^34^ These inversions can be attributed to relaxation effects on the time scale of the geminate SCRP lifetime. Such experiments imply that the ^19^F photo-CIDNP signal intensity of fluorinated amino acids in solution is not necessarily meaningful for comparable experiments of protein-incorporated amino acids. However, using 3-fluorotyrosine, it has already been observed that the ^19^F photo-CIDNP signals of surface-exposed 3-fluorotyrosine in proteins can be recorded in 16 scans (eight of which are dark scans used to subtract the thermal polarization) at a concentration of 1 mM, resulting in a comparable signal intensity as in dark ^19^F NMR spectra recorded using 256 scans.^44^ Our experiments suggest that 4- and 6-fluorotryptophan, combined with fluorescein as photosensitizer, should yield comparable enhancements since DFT-predicted hyperfine couplings are similar in 3-fluorotyrosine and 6-fluorotryptophan.^35^ Our future efforts will focus on demonstrating how this derivative performs in protein photo-CIDNP experiments involving inter- and intramolecular SCRPs.

## Supplementary Material



## Notes

Line broadening in ^19^F resonances following irradiation; ^1^H correlation plots; ^19^F continuous-wave photo-CIDNP; microsecond kinetics of photo-CIDNP polarization; DFT calculations of hyperfine couplings of 4-, 5-, 6- and 7-fluorotryptophan (PDF)
