# Supplementary material for: Fluorinated Tryptophan Derivatives for Photo-CIDNP NMR
Source: J Phys Chem B. 2026 Jun 10;130(30):7621–33. doi: 10.1021/acs.jpcb.6c01954 (PMC13430629; doi:10.1021/acs.jpcb.6c01954)
Supplement: Supplementary file 1 [file jp6c01954_si_001.pdf]

Supporting Information

Fluorinated Tryptophan Derivatives for Photo-CIDNP NMR

Anton Schmidt,[1] Magdalena J. Faber,[1] Audrey Ayekoi,[1] Boris Illarionov,[2] Adelbert Bacher,[3] Markus Fischer,[2] Stefan Weber[1]

[1]*Institute of Physical Chemistry, University of Freiburg, 79104 Freiburg, Germany*

[2]*Hamburg School of Food Science, Institute of Food Chemistry, University of Hamburg, 20146 Hamburg, Germany*

[3]*Department of Chemistry, Technical University of Munich, 85748 Garching, Germany*

Contents

**I.**	**Line Broadening in ^{19}F Resonances Following Irradiation**	**S1**
**II.**	**^{1}H Correlation Plots**	**S4**
**III.**	**^{19}F Cw-CIDNP**	**S5**
**IV.**	**Microsecond Kinetics**	**S7**
**V.**	**Electronic Structure from Tr-CIDNP and DFT Calculations**	**S8**
**VI.**	**References**	**S10**

I. Line Broadening in ^{19}F Resonances Following Irradiation

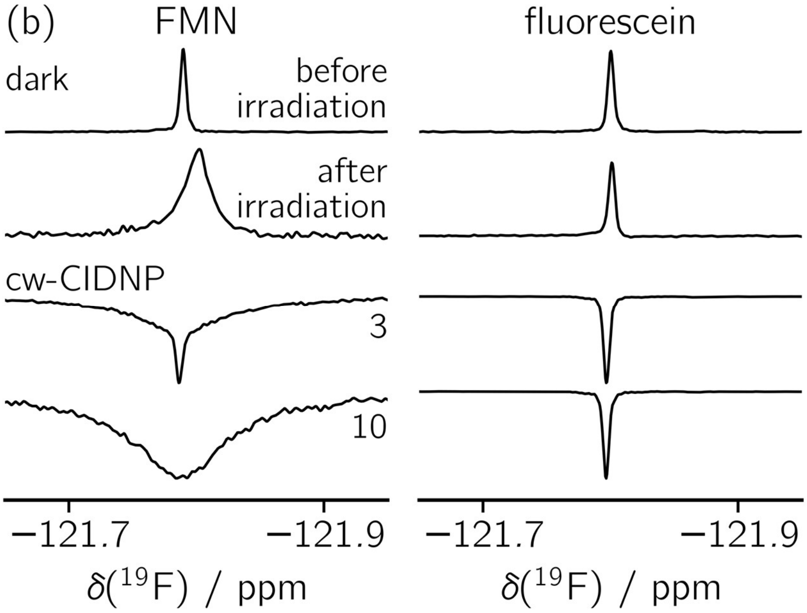

Figure S1: Signals of F6 from 6-fluorotryptophan in a sample containing 2 mM of each 4-, 5-, 6-, and 7-fluorotryptophan, as well as either 200 µM FMN (left) or 100 µM fluorescein (right). The same samples were used as in Figure 6 of the main text. Dark ^{19}F NMR spectra (128 scans, positive amplitudes) were acquired before and after 64 consecutive 16-scan ^{19}F cw-photo-CIDNP measurements. The displayed ^{19}F cw-photo-CIDNP spectra (16 scans, negative amplitudes) are the third and tenth measurement of the 64 measurements.

Instead of one species with a defined linewidth, a mixture of two species with different linewidths is present (see Figure S1). Under prolonged irradiation, the contribution of the resonance with the narrow linewidth disappears, while the contribution of the resonance with the broad linewidth increases. After ten 16-scan photo-CIDNP measurements, the line shape resembles only the broad resonance. Based on this observation, it can be concluded that a second species forms under irradiation in the presence of FMN. This species is either a product of an irreversible photooxidation with the flavin, which occurs between flavins and tryptophan, or of reactions with singlet oxygen or other reactive oxygen species, which are generated by the triplet flavin in the presence of oxygen.[1,2] As a broad resonance was still observed in a degassed sample containing FMN and 6-fluorotryptophan, we hypothesize that the observed photooxidation process is independent of exogenous oxygen. This second species is still redox-active, as evidenced by the appearance of photo-CIDNP polarization. Since a dark ^{19}F NMR spectrum recorded after prolonged irradiation also shows an increased linewidth, it can be concluded that the second species is stable for at least several minutes.

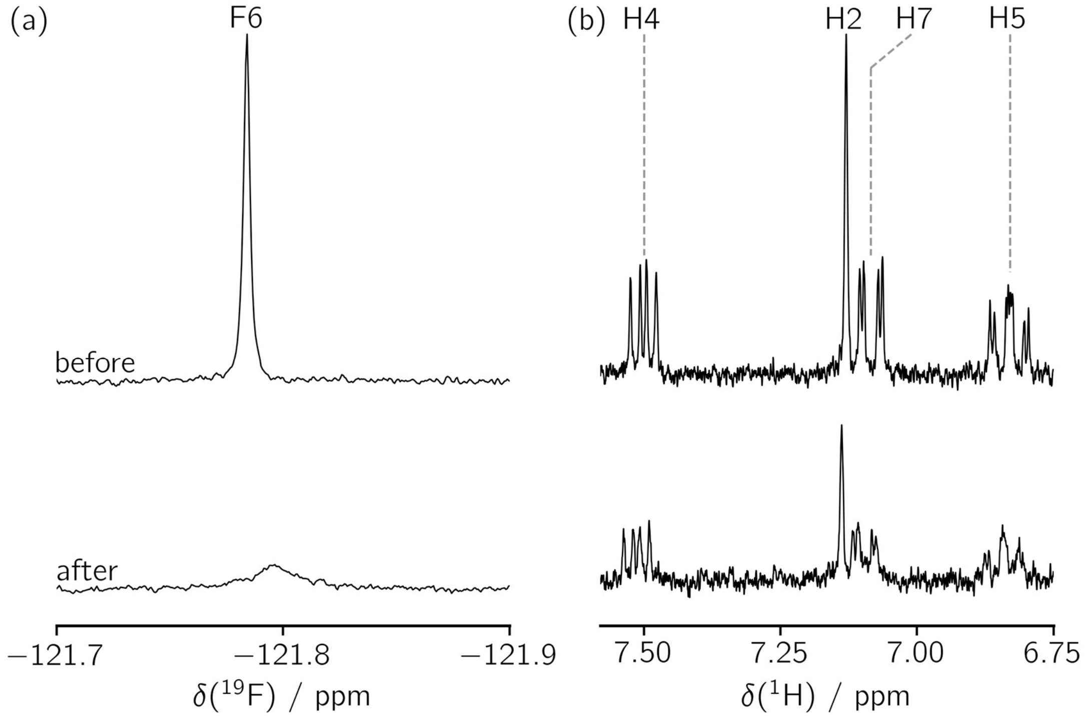

Figure S2: 16-scan (a) ^{19}F and (b) ^{1}H dark NMR spectra of a sample containing 1 mM 6-fluorotryptophan and 200 µM FMN. The noise level is consistent across both the ^{19}F and ^{1}H spectra. All spectra were acquired on the same sample at 7.05 T, before (top) and after (bottom) 64 consecutive cw-photo-CIDNP measurements. The integral ratios after to before were 0.44 (F6), 0.48 (H4), 0.49 (H2), 0.60 (H7) and 0.51 (H5), respectively.

Minimal differences in the chemical shift were observed during the ^{19}F cw-photo-CIDNP measurements. Considering that sample heating caused by sample irradiation can induce chemical shifts in the resonances of analyte[3] and deuterated water,[4] which the spectrometer uses as a lock signal, it is likely that the electronic structure around the fluorine atom of the second species is very similar to that of the respective fluorinated tryptophan derivative. This conclusion is supported by the observation that the ^{1}H NMR resonances of 6-fluorotryptophan did not exhibit significant chemical shifts or changes in the coupling pattern (Figure S2). However, the 6-fluorotryptophan ^{1}H signals were weaker after the cw-photo-CIDNP measurements. This can be attributed to a photochemical reaction between the flavin and the fluorinated tryptophan derivative.[1] Additionally, line broadening was not observed for the ^{1}H resonances. Therefore, the origin of the line broadening is specific to the fluorine nucleus. We conclude that the large CSA of the ^{19}F nucleus, compared to that of the ^{1}H nucleus, is responsible for the difference in ^{19}F and ^{1}H transverse relaxation. However, for aqueous samples at room temperature and magnetic field strengths of 7.05 T, CSA relaxation is usually only relevant for molecules with high rotational correlation times (large

molecules), while dipole-dipole (DD) relaxation is dominant for ^{19}F nuclei in molecules with low rotational correlation times (small molecules).[5] Hence, CSA relaxation and DD-CSA cross-correlation would not be expected to contribute significantly to the transverse relaxation at 7.05 T. Unfortunately, overall low signal intensities and an unstable signal intensity after photo-CIDNP experiments (see Figure 6 of main text), combined with the problem of diffusion from the non-irradiated, fresh sample region from above the fiber tip end into the coil region, did not permit a deeper analysis of the relaxation mechanism. More sophisticated experiments at various magnetic field strengths are necessary to unravel the origin of the increase in transverse relaxation. Therefore, we cannot definitely answer the question of what leads to the broadening of the ^{19}F signal right now. However, fluorescein is a solution to this problem, as its resonance does not broaden under irradiation, and is therefore the superior photosensitizer when a large S/N-ratio is desired.

II. ^{1}H Correlation Plots

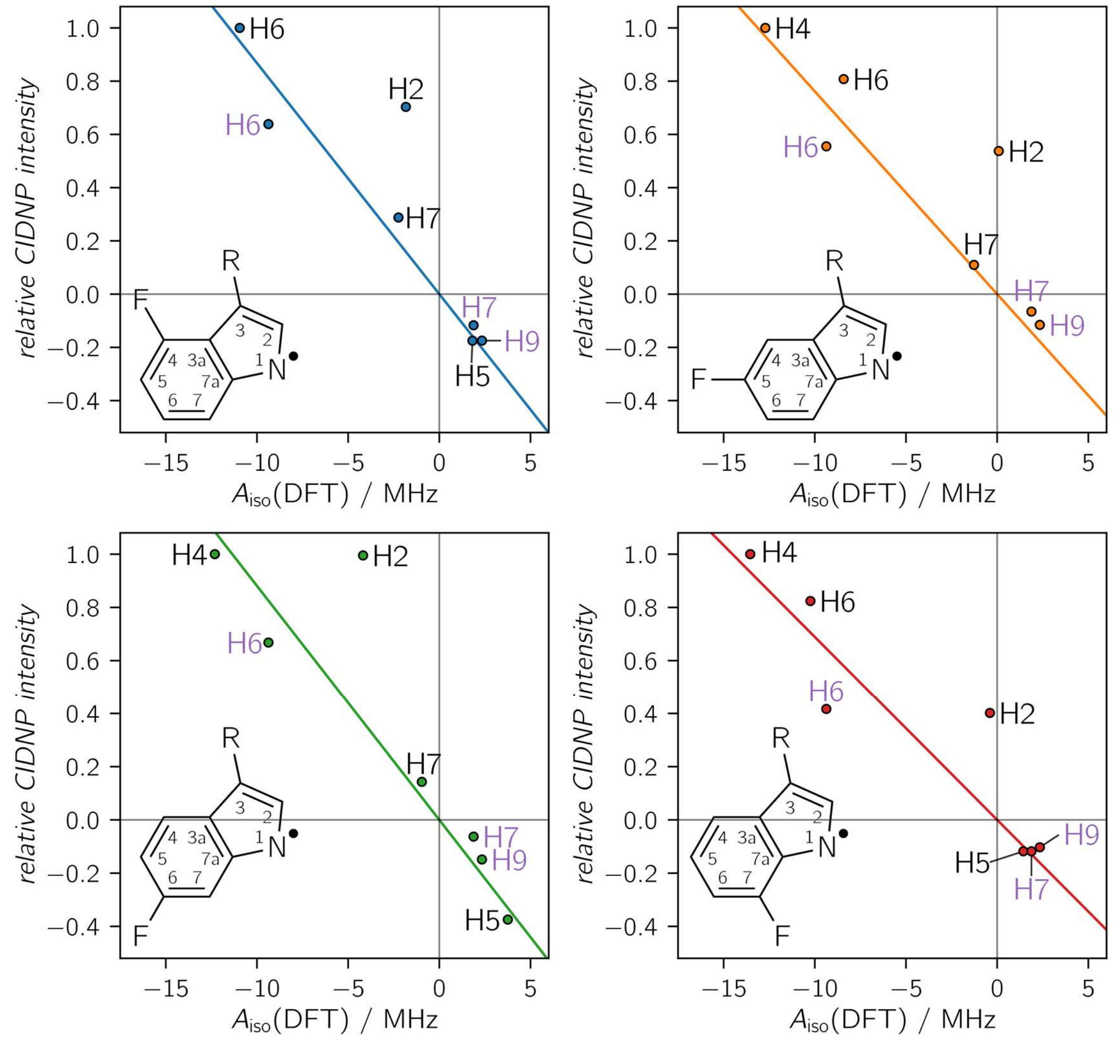

Figure S3: Geminate ^{1}H photo-CIDNP/DFT correlation plots of the neutral radicals of 4-fluorotryptophan (upper left, R^2 = 0.7549), 5-fluorotryptophan (upper right, R^2 = 0.6830), 6-fluorotryptophan (lower left, R^2 = 0.7687) and 7-fluorotryptophan (lower right, R^2 = 0.8340). The geminate ^{1}H photo-CIDNP intensities were multiplied by –1 for protons in 8-fluoro-7,8-didemethyl-FMN to account for Kaptein's sign rule;[6] the labels are shown in purple for easier distinction.

III. ^{19}F Cw-CIDNP

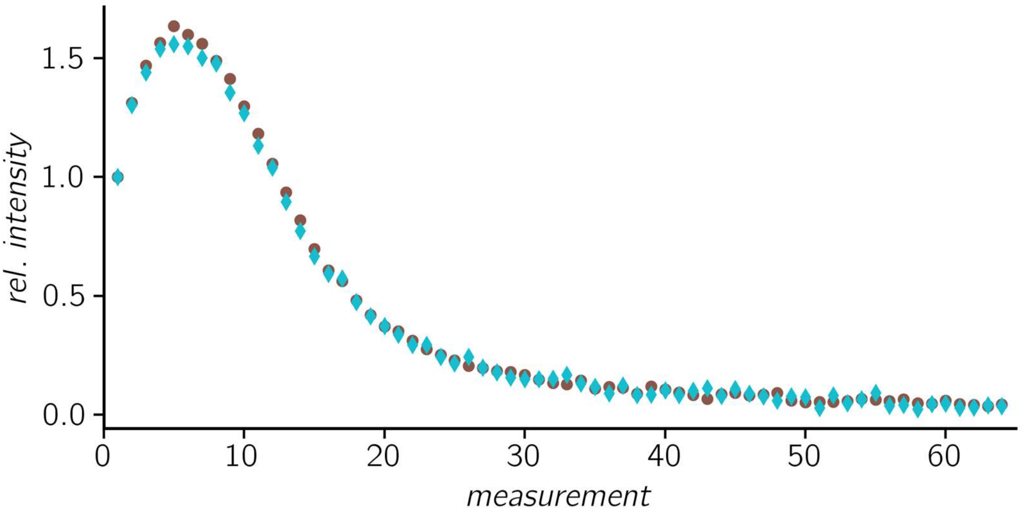

Figure S4: Evolution of the relative signal intensities (integrals) of F4 (cyan diamonds) and F6 (brown circles) in consecutive ^{19}F cw-photo-CIDNP measurements of a sample containing 2 mM of each 4,- 5,- 6,- and 7-fluorotryptophan, as well as 200 µM FMN.

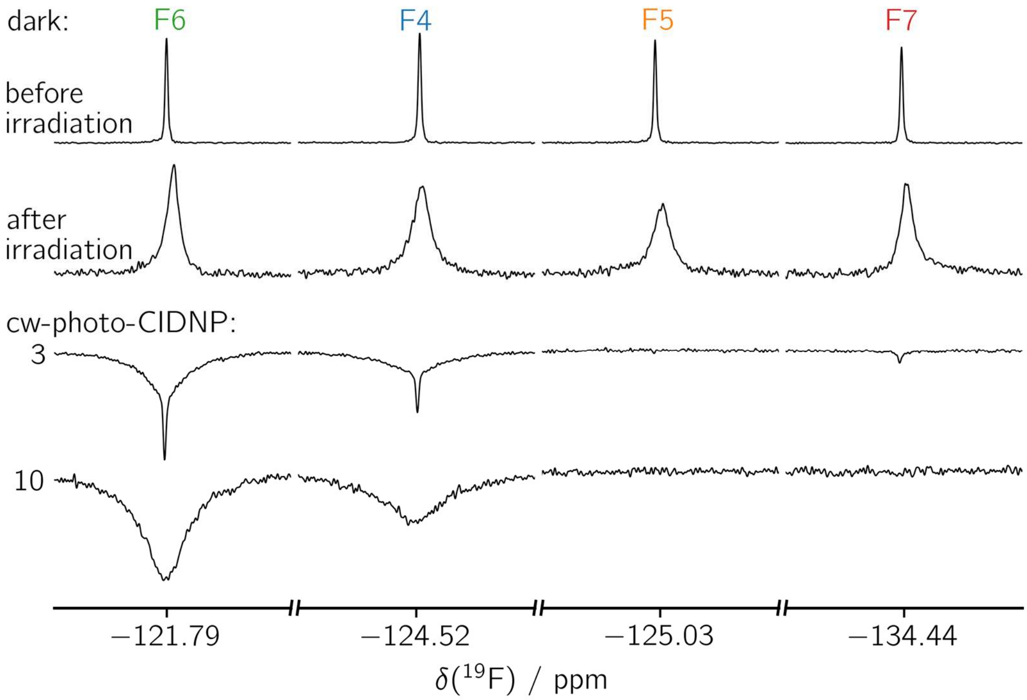

Figure S5: Dark ^{19}F NMR (128 scans) and ^{19}F cw-photo-CIDNP (16 scans) spectra of a sample containing 2 mM of each 4,- 5,- 6,- and 7-fluorotryptophan, as well as 200 µM FMN. The displayed ^{19}F cw-photo-CIDNP spectra (negative amplitudes) are the third and tenth of 64 consecutive measurements. Dark spectra (positive amplitudes) were acquired before and after the ^{19}F photo-CIDNP measurements.

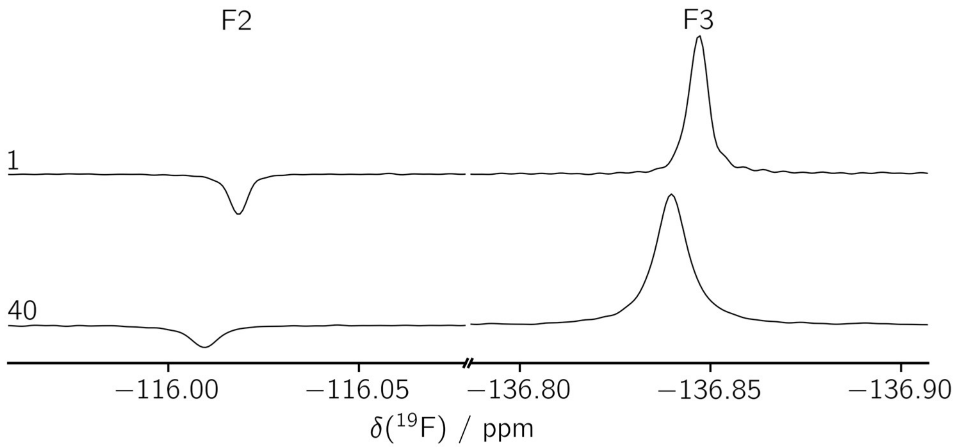

Figure S6: 16-scan ^{19}F cw-photo-CIDNP spectra of a sample containing 1.67 mM 2-fluorotyrosine, 1.67 mM 3-fluorotyrosine and 200 µM FMN. The displayed spectra are the first and 40th of 40 consecutive measurements.

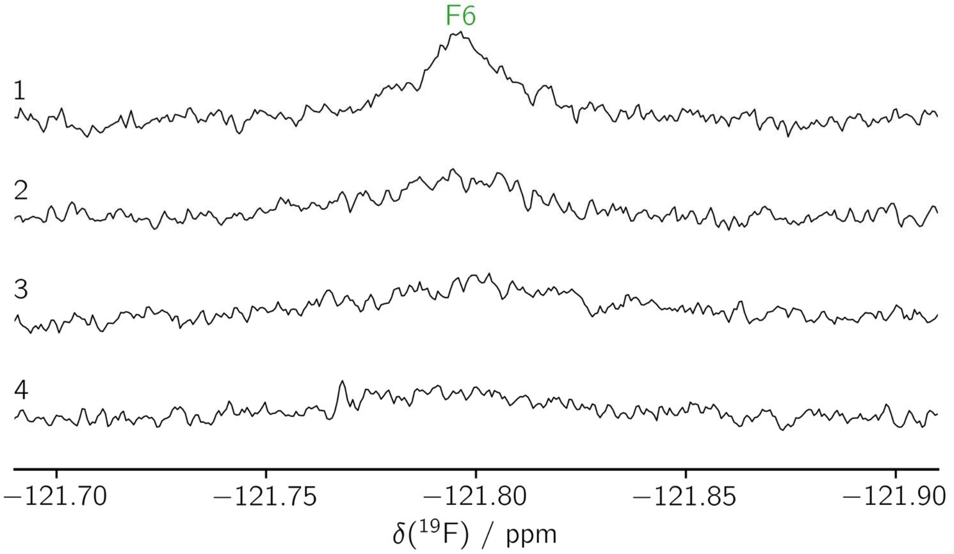

Figure S7: 16-scan ^{19}F dark NMR spectra of a sample containing 1 mM 6-fluorotryptophan and 200 µM FMN. The noise level is consistent across all spectra. All spectra were acquired after 64 consecutive cw-photo-CIDNP measurements. Each spectrum was acquired without further irradiation and with 466 seconds between acquisitions.

VI. Microsecond Kinetics

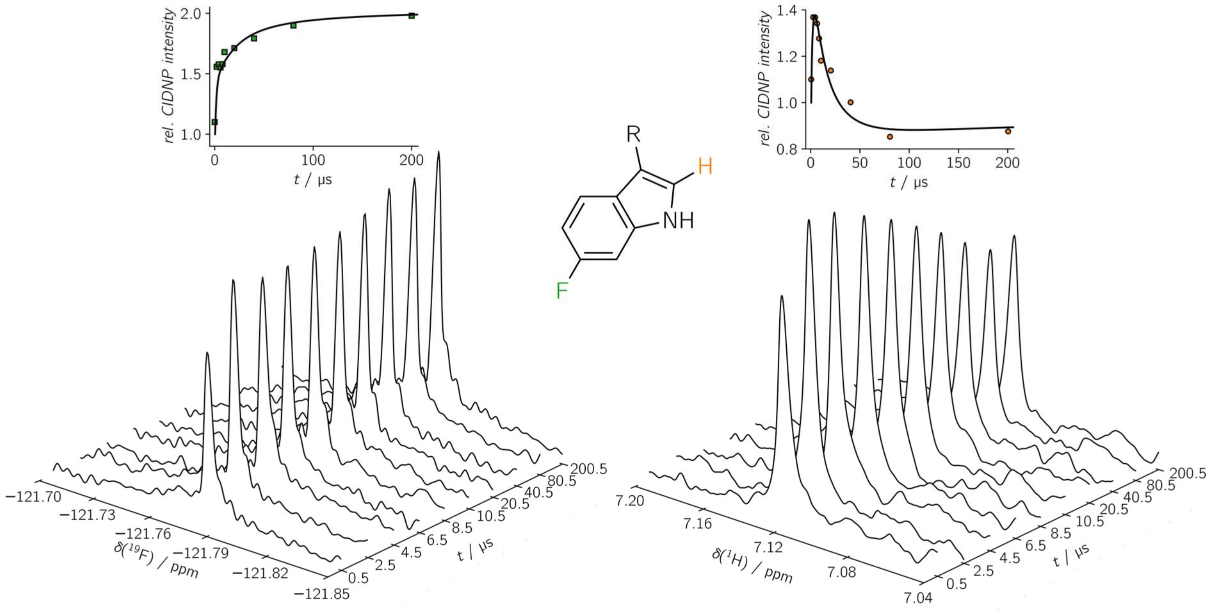

Figure S8: Microsecond kinetics of photo-CIDNP polarization for H2 (orange circles) and F6 (green squares) of 6-fluorotryptophan in the photoreaction with FMN. For a better comparability, the absolute values of the photo-CIDNP signal intensities were evaluated. The 3D plots show the evaluated signals for various delays between laser excitation and sampling pulse. The parameters for the simulation (solid line in 2D plots) were: R_0k_t: 2.8 × 10^5 s^{-1} (both), T_1: 8 µs (F6) and 55 µs (H2). P^G was set to 1, k_{ex} was set to 0 and γ was set to 2.8.

V. Electronic Structure from Tr-CIDNP and DFT Calculations

Table S1: [1]H isotropic hyperfine coupling constants in the anionic 8-fluoro-7,8-didemethyl-FMN radical. Geminate ^{1}H photo-CIDNP intensities were gathered from experiments with either 4-, 5-, 6- or 7-fluorotryptophan (Figure 2 of main text) and normalized with respect to the strongest signal observed, which was either the signal of H4 in 5-, 6- and 7-fluorotryptophan or the signal of H6 in 4-fluorotryptophan (Table S2).

	anionic radical	4-fluoro-tryptophan	5-fluoro-tryptophan	6-fluoro-tryptophan	7-fluoro-tryptophan
Nucleus	A_{iso}/MHz (DFT)	rel. A_{iso} (CIDNP)	rel. A_{iso} (CIDNP)	rel. A_{iso} (CIDNP)	rel. A_{iso} (CIDNP)
H6	–9.37	0.64	0.56	0.67	0.42
H7	2.34	–0.17	–0.12	–0.15	–0.10
H9	1.88	–0.12	–0.07	–0.06	–0.12

Table S2: [1]H isotropic hyperfine coupling constants of aromatic protons in cationic and neutral fluorinated tryptophan radicals. Geminate ^{1}H photo-CIDNP intensities were gathered from experiments with 8-fluoro-7,8-didemethyl-FMN and normalized with respect to the strongest aromatic signal observed. DFT-calculated values were normalized with respect to the strongest coupling obtained for the aromatic protons.

			cationic radical		neutral radical	
	Nucleus	rel. A_{iso} (CIDNP)	rel. A_{iso} (DFT)	A_{iso}/MHz (DFT)	rel. A_{iso} (DFT)	A_{iso}/MHz (DFT)
4-fluoro-tryptophan	H2	0.70	0.79	–9.01	0.17	–1.83
	H5	–0.18	–0.20	2.28	–0.17	1.82
	H6	1.00	1.00	–11.34	1.00	–10.94
	H7	0.29	0.43	–4.93	0.20	–2.24
5-fluoro-tryptophan	H2	0.54	0.55	–8.24	–0.01	0.09
	H4	1.00	1.00	–15.08	1.00	–12.71
	H6	0.81	0.65	–9.82	0.66	–8.42
	H7	0.11	0.14	–2.11	0.10	–1.27
6-fluoro-tryptophan	H2	1.00	1.00	–13.81	0.34	–4.18
	H4	1.00	0.95	–13.08	1.00	–12.31
	H5	–0.37	–0.32	4.48	–0.31	3.76
	H7	0.14	0.17	–2.42	0.08	–0.96
7-fluoro-tryptophan	H2	0.40	0.44	–7.40	0.03	–0.40
	H4	1.00	1.00	–16.71	1.00	–13.54
	H5	–0.12	–0.14	2.40	–0.11	1.44
	H6	0.82	0.70	–11.62	0.76	–10.24

Table S3: ^1H isotropic hyperfine coupling constants of aliphatic protons in cationic fluorinated tryptophan radicals. Geminate ^1H photo-CIDNP intensities were gathered from experiments with 8-fluoro-7,8-didemethyl-FMN and normalized with respect to the strongest aromatic signal observed (see **Table S2**). The absolute values of the isotropic hyperfine coupling constants were calculated from the geminate photo-CIDNP intensities and the slopes in the correlations plots of aromatic protons (4-fluorotryptophan: m = –0.0779 MHz^{-1}, 5-fluorotryptophan: m = –0.0875 MHz^{-1}, 6-fluorotryptophan: m = –0.0734 MHz^{-1}, 7-fluorotryptophan: m = –0.0593 MHz^{-1}; see Figure 3).

	Nucleus	rel. A_{iso} (CIDNP)	A_{iso}/MHz (calc.)
4-fluoro-tryptophan	Hα	0.10	–1.3
	Hβ1	–1.76	22.6
	Hβ2	–2.14	27.5
5-fluoro-tryptophan	Hα	0.06	–0.9
	Hβ1	–1.73	25.6
	Hβ2	–2.30	34.1
6-fluoro-tryptophan	Hα	0.06	–0.8
	Hβ1	–1.66	22.6
	Hβ2	–2.14	29.2
7-fluoro-tryptophan	Hα	0.08	–1.4
	Hβ1	–1.55	26.1
	Hβ2	–1.83	30.9

Table S4: Calculated isotropic g-values of cationic and neutral radicals of fluorinated tryptophan derivatives, as predicted by DFT.

radical	4-fluorotryptophan	5-fluorotryptophan	6-fluorotryptophan	7-fluorotryptophan
cationic	2.00297	2.00266	2.00295	2.00278
neutral	2.00302	2.00285	2.00301	2.00287

Table S5: Hyperfine tensors of the ^{19}F nuclei in cationic and neutral radicals of 4-, 5-, 6- and 7-fluorotryptophan, as predicted by DFT. The hyperfine anisotropy ΔA was calculated using the equation $\Delta A = A_{ZZ} - (A_{XX} + A_{YY})/2$.

	4-fluorotryptophan		5-fluorotryptophan		6-fluorotryptophan		7-fluorotryptophan	
	cationic	neutral	cationic	neutral	cationic	neutral	cationic	neutral
A_{XX}	–27.11	–16.13	2.47	0.18	–31.12	–21.15	–13.22	–5.54
A_{YY}	–37.53	–26.58	–4.29	–4.98	–38.28	–28.05	–14.58	–7.63
A_{ZZ}	184.52	135.38	–25.01	–11.09	183.41	138.79	66.94	28.89
A_{iso}	39.96	30.89	–8.94	–5.30	38.00	29.86	13.05	5.24
ΔA	216.84	156.74	–24.10	–8.69	218.11	163.39	80.84	35.48

VI. References

[1] Connolly, P. J.; Hoch, J. C. Photochemical degradation of tryptophan residues during CIDNP experiments. *J. Magn. Reson.* **1991**, *95*, 165–173.

[2] Huang, R.; Choe, E.; Min, D. B. Kinetics for singlet oxygen formation by riboflavin photosensitization and the reaction between riboflavin and singlet oxygen. *J. Food. Sci.* **2004**, *69*, C726-C732.

[3] Mohanty, S.; Bernstein, H. J. Medium effects in NMR. VIII. Temperature and pressure dependence of ^{19}F chemical shifts in pure CF_4, SiF_4, and SF_6 gases and in gaseous mixtures. *J. Chem. Phys.* **1971**, *54*, 2254–2264.

[4] Hindman, J. C. Proton resonance shift of water in the gas and liquid states. *J. Chem. Phys.* **1966**, *44*, 4582–4592.

[5] Hull, W. E.; Sykes, B. D. Fluorotyrosine alkaline phosphatase: internal mobility of individual tyrosines and the role of chemical shift anisotropy as a ^{19}F nuclear spin relaxation mechanism in proteins. *J. Mol. Biol.* **1975**, *98*, 121–153.

[6] Kaptein, R. Simple rules for chemically induced dynamic nuclear polarization. *Chem. Commun.* **1971**, 732–733.
